# Progress of Epidemics in Forty-One Towns and Districts

**Published:** 1858-07

**Authors:** 


					187
PROGRESS OF EPIDEMICS.
LOCAL REPORTS OF EPIDEMIC AND ENDEMIC
DISEASES
During the Months of March, April, and May, 1858.
Place.
ikSt.Mary,Scilly
Teignmouth -
Portsmouth -
Odiham -
Canterbury -
Chatham
Wandsworth -
Putney -
Up. Holloway
Swansea
Colchester
Aspley Guise -
Saffron Walden
Bedford-
Newpt.Pagnell
Sharnbrook -
Wellingbro' -
Beccles -
Thetford
Stourbridge -
Dudley -
Barrowden
Wisbeach
East Dereham I
Pontesbury -
Nottingham -
Burton-on Trt.
Oswestry
Wrexham
Hawarden
Lincoln
Alford -
Gainsborough
Liverpool
Warrington -
Wigan -
Bolton -
York
Wst. Auckland
Bothbury
Lerwick
County.
Cornwall
Devonshire -
Hampshire -
Hampshire
Kent
Kent
Surrey -
Surrey -
Middlesex
Glamorgansh.
Essex -
Bedfordshire -
Essex -
Bedfordshire -
Buckinghams.
Bedfordshire -
Northamptons.
Suffolk -
Norfolk -
Worcestersh. -
Staffordshire -
Rutland
Cambridgesh.
Norfolk -
Shropshire
Nottinghamsh.
Staffordshire -
Shropshire
Denbighshire -
Flintshire
Lincolnshire -
Lincolnshire -
Lincolnshire -
Lancashire
Lancashire
Lancashire
Lancashire
Yorkshire
Durham
Northumberld.
Shetland Isles
Lat.
49.50 N.
50.32 N.
40.58 N.
51. 8 N.
51.17 N.
51.21 N.
51.28 N.
51.28 N.
51.32 N.
51.38 N.
51.54 N.
52. 1 N.
52. 3 N.
52. 8 N.
52.10 N.
52.12 N.
52.20 N.
52.25 N.
52.26 N.
52.28 N.
52.30 N.
52.34 N.
52.39 N.
52.40 N.
52.43 N.
52.50 N.
52.53 N.
52.53 N.
53. 2 N.
53.11 N.
53.12 N.
53.15 N.
53.23 N.
53.24 N.
53.25 N.
53.32 N.
53.35 N.
53.58 N.
54.45 N.
55.25 N.
60. 9 N.
Long."
6.24 W.
3.29 W.
6. 6 W.
1. 3 W.
1. 4 E.
0.14 E.
0. 7 W.
0. 8 W.
0.03 E.
3.50 W.
0.52 E.
0.37 W.
0.12 E.
0.51 W.
0.42 W.
0.40 W.
0.40 W.
1.48 E.
0.45 E.
2.32 W.
2.10 W.
0.38 W.
0/ 5 E.
0.57 E.
2.50 W.
1.10 W.
1.53 W.
2.59 W.
3. 1 W.
3. 2 W.
0. 5 W.
0. 6 E.
0.47 W.
2.59 W.
2.32 W.
2.33 W.
2.19 W.
1. 3 W.
1.40 W.
1.50 W.
1. 8 W.
Observer.
J. G. Moyle, Esq.
W. C. Lake, Esq.
Alfred Jackson, M.D.
J. Mclntyre, M.D.
G. Rigden, Esq; *
F. J. Brown, M.D.
G. E. Nicholas, Esq.
R. H. Whiteraan, Esq.
W. B. Kesteven, Esq.
W. H. Michael, Esq.
Henry Laver, Esq.
J. Williams, M.D.
T. Spurgin, Esq.
H. Stear, Esq.
T. H. Barker, M.D.
G. O. Rogers, Esq.
R. S. Stedman, Esq.
B. Dulley, Esq.
W. E. Crowfoot, Esq.
H. W. Bailey, Esq.
A. Freer, Esq.
J. H. Houghton, Esq.
H. J. Swann, Esq.
W. H. Hole, Esq.
J. Vincent, M.D.
Wm. Eddowes, Esq.
T. Robertson, M.D.
S. Thomson, M.D.
P. Cartwright, Esq.
E. Williams, M.D.
T. Moffat, M.D.
S. Lowe, Esq.
R. U. West, M.D.
D. Mackinder, M.D.
Thos. Bickerton, Esq.
C. N. Spinks, Esq.
Jephtha Hussey, Esq.
W. H. Pendlebury,Esq.
W. Procter, Esq.
G.Todd, Esq.
E. C. Summers, Esq.
G. W. Spence, M.D.
QUARTERLY STATEMENT?No. XIV.
[ The dates denote that the disease appeared in the weeks then ending."]
SCARLET FEVER.
Portsmouth?April 23
Canterbury?May 21-28
Chatham?May 21-28
Putney?Every week
Holloway?March 26, April 30
Swansea?Every week
Colchester?May 7-28
Aspley Guise?April 9-16, May 21-28
Bedford?May 7-21
Beccles?All Mar., April 2-9, all May
Stourbridge?Mar. 26, Apl. 2-9, May 7,
Dudley?Mar.5,19,Apl.9,23-30 [21-28
Barrowden?Mar. 12, 26, April 9, 23
188 PROGRESS OF EPIDEMICS:
Wisbeach?March 5-12
Nottingham?Mar. 5, April 30, May 7
B'irton-on-Trent?M ar. 12, Apl.3 0,M ay
Oswestry?April 2 [28
AVrexham?All March
Hawarden?March 5-12
Lincoln?April 23-30, all May
Gainsborough?Every week
Liverpool?Every week
Warrington?Every week
Wigan?March 26, April 16, May 7
Bolton le-Moors?AH Mar., April 23-
30, all May
York?Mar. 26, A pi. 0-16,30, May 7-21
MEASLES.
Portsmouth?Mar. 5-19, Apl. 2-9, May
Canterbury?Every week [4, 21
Chatham?Every week
Putney?March 19-26, April 30
Swansea?May 7-28
Colchester?Every week
Beccles?All March and April
Stourbridge?March 12-19
Barrowden?March 19-26, April 9
Nottingham?April 9-16
Wrexham?Every week
Liverpool?Every week
Warrington?May 7
Bolton le-Moors?Mar. 19-26, Apl.2-9,
York?Mai .5-12,26, Apl. 9 [30,all May
West Auckland?Every week
SMALL POX.
Portsmouth?March 12, May 14
Canterbury?All May
Chatham?April 9-30, all May
Swansea?Every week
Colchester ?Mar. 19-26, all Apl. & May
Bedford?All March, April 2-9
Stourbridge?March 5, April 2-9
D u d 1 ey?M arch 19
Barrowden?Every week
Wisbeach?Every week
Pontesbury?April 16-30, May 7-21
Nottingham?March 26
Burton-on-Trent?March 12, Apl. 2-9
Oswestry?Every week
Lincoln?May 14-28
Liverpool?Every week
Warrington?All March and April
Wigan?Mar.5,19-26,Ap.16,30,all May
Bolton-le Moors?All March, April 16-
30, May 7-14
York?March 12, 26, April 2, 16, 30
Lerwick?May 21-28
HOOPING COUGH.
St. Mary's, Scilly?April 9-30, all May
Portsmouth?April 16, May 14
Canterbury?Every week
Chatham?May 7, 21-28
Wandsworth?April 16, 30, May 14
Hollo way?March 5 12
Wanstead?April 2-10, May 7
Swansea?April 16-80, May 7
Colchester?Every week
Newport Pagnell?March 5-19
Beccles?April 16 23, May 14 28
Ali'ord?Every week
Gainsborough?Every week
Liverpool?Every week
Warrington?All April and May
Wigan?March 19-26, April 1(5, 30
Bolton-le Moors?All Mar.,Apl.2,9-16,
York?Apl.16 30,May 14-21 [Alav7-21
CROUP.
Teignmouth?April 9
Odihara?March 5-12
Canterbury?Mar. 5, April 30, May 28
Chatham?Mar. 19, April 2-9, May 28
Wandsworth?March 12
Putney?March 5, April 30
Hollo way?March 12, April 16
Swansea?March 5-12, April 23 30
Saffron Walden?April 16, May 28
Newport Pagnell?April 23, May 28
Beccles?March 5-12
Thettbrd?April 9, 23
Stourbridge?Mar. 26, Apl. 23-30, May
Dudley?March 12 [7-21
Wisbeach?March 5-19
Pontesbury?March 12, April 30
Burton-on-Trent?May 21
Lincoln?March 5-19
Alford?March 26, May 14
Liverpool?All Mar. & Apl., May 7, 28
Warrington?April 2
Wigan?April 16, May 21
Bolton-le-Moors?Mar. 19-2f>, April 2,
York?May 14-28 [23-30, May 14-28
CATARRH.
St. Mary's, Scilly?April 30, all May
Teignmouth?AllMar.&Apl.,May7,21
Portsmouth?March 5-19
Odiham?All March
Chatham?Mar. 12-19, Apl. 16-30, May
Wandsworth?Every week [21-28
Putney?All March and April, May 7
Ilolloway?March 12
Swansea?Mar. 5, Apl. 16-30, all May
Colchester?All March and April
Aspley Guise?March 26, April 9
Saffron Walden?Mar.5-19,Apl. 2, May
Bedford?April 23-30 [7
Newport Pagnell?Mar. 5-19, Apl. 16-
30, all May
Wellingborough?All Mar., April 2-16
Beccles?All March, May 7-21
Thetford?Every week
Stourbridge?April 9-16
Dudley?March 12, April 16
Barrowden?Match 5, April 9
LOCAL REPORTS. 189
Wisbeach?AH March, April 2-9
Pontesbury?March 12, May '21
Nottingham?All March
Oswestry?March 5
Wrexham?Mar. 12-26, April 2,16-30
Alford?May 14
Gainsborough?Every week
Liverpool?Every week [May 7-14
Bolton-le-Moors?All March, Apl. 2-9,
Rothbury?Mar. 26, all April and May
Lerwick?Mar. 5-12, 26, April 16, 30,
May 7-21
INFLUENZA..
St. Mary's, Scilly?All Mar., Apl. 2-16
Teignmouth?Mar. 5-19, Apl. 16, May
Odiham?April 9 [7-21
Chatham?Mar. 5,26, Apl. 16, all May
Wandsworth?March 26
Wanstead?March 5-12
Saffron Walden?Mar.l2-2R,Ap.2,May
Newport Pagnell?Every week [21-28
Wellingborough?Every week
Beccles?May 21 28
Thetford?All Mar*, Apl. 9 30, May 7-
Stouibridge?April 9-16 [14, 28
Pontesbury?March 5-19
Nottingham?March 5-19, May 21
Oswestry?April 30, all May
Wrexham?April 23-30, May 14-21
Lincoln?All March, April 2-16
Alford?March 12-26
Liverpool?Every week
Warrington?March 26, April 9
Bolton le-Moors?March 19-26, April
2,23-30, May 7
Lerwick?Marc h 12, April 16
ERYSIPELAS.
Portsmouth?April 30, May 14
Odiham?March 26
Canterbury?March 5, 19
Chatham?May 7, 28
Wandsworth?April 30, May 14
Putney?May 28
Wanstead?May 7
Swansea?March 5, all May
Saffron Walden?March 5, May 28
Newport Pagnell?Mar. 12-26, Apl. 2-
23, May 28
Wellingborough?March 5-19
Thetford?March 12, April 2
Stourbridge?March 12, 26, April 2
Dudley?March 19, April 2-9
Barrowden?May 21
Wisbeach?March 26, April 2-9
Pontesbury?All Mar., Apl. 9-30, May
Nottingham?April 16 [7-21
Oswestry?March 5-12
Wrexham?March 26, April 2-9
Gainsborough?April 9-16
Liverpool?All Mar.,Apl.9,23-30,May7
Warrington?March 26, May 21
Bolton-le-Moors?March 12 26, April
16-30, May 7-14
CHOLERA (ENGLISH).
Stourbridge?April 2
Liverpool?March 5, April 30
AGUE.
Odihara?April 2-9
Canterbury?Mar. 12-19, all Apl., May
Chatham?Every week [21
Colchester?Every week
Saffron Walden?Mar. 12-19, April 2-9
Bedford?Every week
Newport Pagnell?April 30, all May
Thetford?Mar.12, Apl. 9,23-30, May7-
Dudley?Mar. 12, Apl. 30 [14, 28
Barrowden?March 12-26, May 7-14
Wisbeach?Every week
Lincoln?Every week
Alford?April 9, 23, all May
Gainsborough?Mar. 12-19, Apl. 2-23
Liverpool?May 14
Warrington?April 23 30, May 7-14
REMITTENT FEVER.
Teignmouth?March 26, May 14-21
Odiham?April 30, May 14
Canterbury?Mar.12, Apl. 23, May 7-14
Wandsworth?April 30
Putney?April 16, 30
Newport Pagnell?April 30, all May
Thetford?March 5, April 23, May 28
Stourbridge?March 19-26, May 7,28
Wisbeach?All April, May 7
Oswestry?March 5-12, May 7-21
Alford?Every week
Liverpool?Every week
Warrington?April 9
Wigan?April 30, May 7-14
Bolton-le-Moors?March 19-26, April
2, 30, all May
York?April 9-16, 30, May 14
Rothbury?March 5 19, May 7-14
DIARRHOEA.
St. Mary's, Scilly?May 14-21
Portsmouth?April 23
Odiham?March 26, April 9, 23-30
Canterbury?May 14-28
Chatham?Mar, 12, April 2,30, May 14
Wandsworth?All Mar., Apl. 9-30, May
Putney?All Mar., May 28 [21-28
Hollovvay?May 14
Swansea?Mar.5-12,26, Apl. 2-23, May
Colchester?May 14-28 [14-28
Ashley Guise?May 14-28
Saffron Walden?April 30, May 14
Bedford?-May 21-28 [May 7
Newport Pagnell?Mar. 12-19, Apl. 30,
Wellingborough?April 30, all May
Beccles?April 16-30 [14, 28
Thetford?Mar.5,19-26, all Apl.,May 7-
190 PROGRESS OF EPIDEMICS I
Stourbridge?March 12-19, April 16
Dudley?Every week
Barrowden?March 5, 26, April 9
Wisbeach?May 14-28
Pontesbury?All Mar., April 2-23, all
Nottingham?May 7-14 [May
Burton-on Trent?April 9
Oswestry?Mar. 5 12, all April & May
Wrexham?March 26, April 2-16, May
14-28
Hawarden?Mar. 12,26, April 2-16,30,
Liverpool*?Every week [May 7-21
Warrington?May 14-28
Wigan?April 9, 23, May 7-14
Bolton-le- Moors?Mar.26,Apl.2,23,all
York?April 30, May 14-28 [May
Rothbury?Mar.l2-19,Apl. 2-9, May 28
Lerwick?March 26, April 2-16
DYSENTERY.
Teignmouth?Mar. 26, April 9-16, 30,
Odiham?May 21 [May 14
Chatham?April 30
Wandsworth?Mar. 19, April 9, 23-30,
Saffron Walden?Mar. 5 [May 14-21
Newport Pagnell?April 16
Wellingborough?May 14-28
Dudley-?March 12
Pontesbury?April 23-30
Wrexham?Mar. 26, Apl. 2-16, May 14-
Hawarden?March 19 [28
Alford?March 19-26, May 14-28
Gainsborough?Every week
Liverpool?All Mar.,Apl.] 6-30, all May
Warrington?All May
Wigan?April 16
Bolton-le-Moors?March 19-26, April
2, 16-23, all May
York?April 23, May 7, 21-28
TYPHUS.
Teignmouth?Mar.26, Apl.30, May 14-
Portsmouth?March 12 [21
Canterbury?March 26, April 2-16
Chatham?May 21
Wandsworth?Mar. 5,19-26, all April,
Putney?All Apl., May 21 [May 7-21
Saffron Walden?April 2-16
Bedford?March 5, 19, April 16-30
Newport Pagnell?Mar. 12-26, all Apl.
Thetford?April 2-16, May 21
Stourbridge?April 16 23
Dudley?Every week
Wisbeach?April 30, all May
Wrexham?All March, April 2-16
Alford?Mar. 5-12, April 23-30, May 7
Liverpool?Every week
Wigan?March 26, April 9, 30
Bolton-le-Moors?March 26, April 2-
16, 30, all May
West Auckland?All March and April
Rothbury?March 5-19
PUERPERAL FEVER.
Teignmouth?May 7
Canterbury?April 9
Chatham?May 21
Wandsworth?March 26
Beccles?March 5-12 [May 7-14
Bolton-le-Moors?Mar. 19-26, Apl. 30,
DIPHTHERITE.
Canterbury?All Mar., Apl. 23-30, all
Portsmouth?April 16 [.May
Colchester?April 23-30, all May
Lincoln?'May 21-28
CONTINUED FEVER.
Lerwick?April 23
TYPHOID FEVER.
Colchester?Every week
carbuncle.
Portsmouth?April 9, May 7
Canterbury?Every week
Chatham?March 5
Wandsworth?March 5, May 7
Holloway?March 5-12
Wanstead?April 23, May 14
Swansea?March 5
Saffron Walden?March 19
Bedford?April 9
Wellingborough?May 14-28
Thetford?April 16, May 14
Stourbridge?April 30
Oswestry?May 14-21
Liverpool?March 19-26, April 2
Warrington?April 16 [May 7-14
Bolton-le-Moors?Mar. 26, Apl. 2 23,
West Auckland-?April 23-30
MUMPS.
Lerwick?April 2
ADDITIONAL OBSERVATIONS.
St. Mary's, Stilly.?Mr. Moyle writes : " The quarter ending
May 28th has been characterised by the existence of three epi-
demics in these islands : influenza all March and the first nine
days of April; hooping cough all April and May, and still con-
tinuing ; varicella prevailing all May and still spreading. No
death occurred from influenza; nor has any happened from
LOCAL REPORTS. 191
hooping cough, although some of the cases have been severe ;
their number has been 80. The varicella was ushered in in
ohe ease by convulsions ; but the child recovered. In April and
May severe cases of catarrh occurred* principally in those
families where hooping cough was present. I can scarcely call
this catarrh epidemic. There were twelve deaths during the
quarter, viz:?
No. Cause of Death. Sex. Age.
1. Senile gangrene    M. 62
1. Phthisis   M. 23
1. Apoplexy  E. 66
1. Softening of brain : dis-
eased spine several years F. 28
1. Infantile convulsions .... 6 mo.
1. Chronic bronchitis  M. 73
>To. Cause of Death. Sex. Age.
1. Cancer of breast  F. 67
1. Pneumonia   F. 71
1. Paralysis    F. 78
1. Chronic dysentery   M. 69
1. Debility from age   M. 88
1. Valvular disease of heart F. 59
<( The weather during the whole quarter has been fine and dry,
7'65 inches of rain only having fallen during the three months ;
we had twenty-two fine days in March. The average of ozone
was one for March ; three for April; and five for May. The
prevailing winds in March were east; in April, west; May
was on the whole stormy, with the winds from the north-west.
There was no epidemic among the cattle, and the crops look
well; the early potatoes have turned out well?a good average
crop." ? ? ; , 1 , -
Teignmouth.?Mr. Lake writes: " The coldest part of the
winter was the time intervening between February 25 and
March 5 ; the temperature then rose suddenly and considerably,
but its daily range was unusually great all through March.
The temperature was very variable during the first half of
April, and on the whole low; it then rose till the 20th of the
month, declined from that date till May 3d; rose gradually, but
to no great extent, through that month, and increased some-
what suddenly towards its close. The barometric pressure was
low in the early part of March, high in the middle of the month,
and from that time variable till the end of the quarter. The
degree of humidity for March was about the average for April
alone ; for May, below the average. During the first week in
March snow fell. 7*137 inches of rain were measured as hav-
ing fallen during the quarter. Of these 0*393 of rain and
melted snow fell between March 2 and 14c; 3 851 from March
30 to April 10 ; 0769 between April 24 and May 4 ; 1984
from May 12th to 24th. The wind during the quarter blew
principally from an easterly direction, except from March 6th
to the 16th, when it blew from north or north-west for a few
days in April and during the middle of May, when it was
westerly or south-westerly. A good deal of ozone existed in
192 PROGRESS OF EPIDEMICS :
the first half of March, little in the latter half It was de-
veloped in considerable quantity during the first part of April,
then only moderately and variably till May 8th, from which
date till the end of the month it again was freely developed.
Diseases affecting the .respiratory organs (excluding influenza)
had largely predominated over those affecting the digestive
system during the last quarter ; during the quarter now ended
the numbers of each class were pretty equal. Influenza showed
itself mostly during the commencement and the close of the
quarter. The following appeared, in the cases that came under
my notice, the most remarkable features of disease during the
quarter:?The prevalence of dysentery, ordinarily commencing
with diarrhoea, as a result of exposure to cold and wet, appa-
rently of the nature of a catarrh of the large bowels, and usually
yielding readily to Dover's powder and fomentation : the not
unfrequent occurrence of cases of urethral and vesical, and also
of uterine irritation, the result of cold, and of a rheumatic or
catarrhal character ; the uterine affection being amenable with-
out much difficulty to Dover's powder and fomentation ; the
vesical or urethral affection being sometimes equally amenable
to the same treatment, but often much more obstinate in its
character : a tendency of the febrile attacks of children to run
into active abdominal inflammation; and an unusual tendency
to the occurrence of delirium of an aggravated character in
febrile and inflammatory affections."
Canter-bury.?Mr. Eigden writes : " During the last three
months several cases of diphtheria have presented themselves.
Many were of a very severe character, and five proved fatal;
viz., two in March and three in April. The disease has been
mostly confined to children and young persons under twenty-
five years of age. Measles has been very prevalent, and four
cases have proved fatal; viz., three in April and one in May.
A few cases of smallpox have been seen, three of which have
fallen under my observation ; they were all in one family; the
disease supposed to have been introduced from Deal, a town
about eighteen miles from here. The first two cases were in
a servant and infant, attacked within two days of each other.
The servant had been vaccinated, and had the variola in a
modified form. The infant, aged seven weeks, not vaccinated,
had the disease in a confluent form, and died on the tenth
day. The mother of the infant was afterwards attacked ; she
had been vaccinated in infancy, and had the variola in a mo-
dified form. All the other inmates of the house had been vac-
cinated, and escaped the contagion. Hooping-cough has been
very general, particularly among the poorer classes. Ague and
LOCAL REPORTS. 193
intermittent forms of disease have been more freqnent than they
were a few years since. Furiinculoid diseases have been com-
mon. From a population of J 8,398 inhabitants, there were 58
deaths registered in March, 43 in April, and 39 in May. Of
these numbers, 24 died from zymotic diseases/'
Chatham.?Dr. Brown says : " Measles have prevailed to a
greater extent during the last three months than has been
known for many years. The disease has proved fatal in
several instances. One of the sequelse noticed in this epi-
demic has been erythema nodosum with scanty renal secre-
tion. Iron, with saline medicines, and especially change of air,
proved efficacious. Ague in various forms continues; one child,
aged eight years, died of it in May. He had head symptoms.
The case was registered "Ague," 17 days; "Cephalitis," 14
days. Phlegmasia dolens occurred in the week ending April
30th, and metria (of adynamic type) in the week ending May
21st. There have been many cases of stomatitis and a few of
diphtheria during the three months. Diphtheria has not ap-
peared in a fatal form. The small number of cases of continued
fever is worthy of note. There were many cases of febricula
(synocha)."
Wandsworth.?Mr. Nicholas says : " During the quarter
just ended, as in that preceding it, the most prevalent diseases
were those of the respiratory organs, which with diarrhoea,
dysentery and continued fever, prevailed during the whole
period. In 327 cases of pauper sickness coming under treat-
ment, the following were the relative proportions of these
diseases :?Continued fever formed one-sixteenth ; diarrhoea
and dysentery, one-twelfth ; while diseases of the respiratory
apparatus constituted one-fourth of the whole. There has
been an almost entire absence of the exanthemata."
P utney.?Mr. Whiteman writes: " There has scarcely been
known a period during which scarlatina has been either so
prevalent or so persistent in the district as in the past quarter.
Very few families have entirely escaped the epidemic, but the
malady has been happily of a mild type, resulting in death in
but two instances only. A severe case of diphtheritis, fatal in
a few hours, was treated in a child at the early part of the
quarter. The throat affection and the effusion of coagulable
lymph were preceded by a slight patchy eruption, somewhat re-
sembling that of scarlatina, but differing in many respects
from the ordinary appearances of the true exanthem. A rather
severe form of diarrhoea prevailed in March, and two or three
cases of fever of a typhoid character were treated during the
VOL. IV. O
194 PKOGRESS OF EPIDEMICS:
following month. Catarrh and bronchitis prevailed extensively
during the easterly winds of March and April/'
Swansea.?Mr. Michael writes : " This quarter has been
marked by the prevalence of epidemic disease. Smallpox has
been especially very prevalent chiefly among the children of
the very poorest inhabitants living in the most densely popu-
lated portions of the town. But no place has been free from its
visitations ; the breezy hill side, with its tidy cottage or com-
modious farm house, has been as prone to its visits as the most
dirty squalid abode of poverty in a back court. Scarlatina has
been generally very mild; in few cases fatal or complicated.
Measles has lately commenced, but the cases I have seen have
not been severe. In one case the eruption of measles was dis-
placed by smallpox the day after the appearance of the first
disease/'
Colchester.?Mr. Laver writes: " The cases of smallpox were
the most important this quarter ; they occurred principally as
isolated cases, and have not been very fatal. Typhoid fever,
although occurring during the whole quarter, has not been very
general. Diphtheria has occasionally appeared, and in my own
practice has been fatal in two out of three cases. Vegetation
has appeared healthy/'
Bedford.?Dr. Barker writes : " Ague has been present in
this district throughout the quarter, although the cases have
not been numerous/'
Thetford.?Mr. Bailey sends the following quarterly report:
?" March.?This month may be considered peculiar from the
extreme fluctuation of the atmospheric pressure, the range
being 1 '6 ; the maximum being on the 22nd, and the lowest
on the 6th, when we experienced a considerable gale of wind.
The amount of rain during the month was *67 of an inch ; and
the mean of the dew point 36, being 8? below the mean of
temperature. At the commencement of the month the tem-
perature was low, only on one day reaching as high as 40?,
and the nights were very severe; on the grass it was as low
as 18?, and three feet from the ground, 20?. On the 13th, a
sudden increase of temperature took place beyond temperate ;
it rose to the maximum degree of 63 on the 24th, and the
lowest during the day was 26?, making a range of 37?. This
sudden vicissitude gave rise to numerous cases of catarrh and
influenza among all grades of society. Pneumonic and pleur-
itic affections were more than usually prevalent, as were also
rheumatic fever and ague. Diarrhoea was also epidemic, in
some cases being very severe, producing the greatest prostra-
tion. One case of fever, commencing with diarrhoea, occurred;
LOCAL REPORTS. 195
it might be called enteric typhus, running a course of three
weeks, leaving anasarcous swellings and excessive debility.
The winds being most prevalent from the N. and N.E. in the
early part of the month, and from the imprudent exposure of
children, numerous cases of bronchitis came under notice.
Many cases terminated fatally; the disease also attacked
adults. Shingles attacked two children in different families ;
they were under three years of age, and ultimately did well.
One case of phthisis pulmonalis terminated fatally in a female
who was predisposed to the disease; her death might have
been accelerated by the sudden change of temperature. A
fatal case of enlargement of the liver, with ascites, occurred in
a female of very abstemious habits, who had been under no
medical treatment until the last week, and not even confined
to bed until two days before death. The casual, diseases
of the month under notice were?abortion, psora, impetigo,
haemorrhoids, procidentia uteri, paralysis, hysteria, hyper-
trophy of the heart, and hydrocele. The diseases of cattle
were more numerous than usual; they were?sore throat,
enteritis, pleuropneumonia, colic, and rheumatism. With
cows, many fatal cases occurred in their calving. The sheep
were very healthy; and the fall of lambs was abundant, with
very few losses. Vegetation was extremely backward, and
the later frosts destroyed fruits which were more forward.
"April.?The barometrical range of this month was less
fluctuating, the range being 1 *2; but the temperature in its
range was more singular for this month, deviating consider-
ably during the first fortnight, and afterwards assuming a
summer temperature. The maximum was on the 16th at
72?, the minimum on the 13th at 35?; the range being 37?.
The dew point was 44?. The diseases arising from the sudden
changes of temperature were chiefly influenza, quinsy, and
among children, congestions of the pulmonary organs. These
were most prevalent during the early part of the month. On
the sudden transition of temperature, diarrhoea became more
prevalent, attacking the rich as well as the poor. Ague and
typhoid fever attacked the working-classes, and especially
those who were deprived of the common necessaries of life.
Rheumatism frequently came under treatment, and pleuro-
dynia was more frequent than I had observed for some months.
One fatal case of croup took place this month, in a child about
ten months old ; it was evidently occasioned by exposure to
the severe cold and frosty nights. With the exception of
influenza, catarrh, and diarrhoea, we had no specific epidemic,
so that the month may be considered as having been more
o 2
196 PROGRESS OF EPIDEMICS:
healthy. The casual diseases of the month, and under treat-
ment, were haemoptysis, ascites, jaundice, asthma, convulsions,
fistula in ano, gout, and furunculus. Upon inquiry of the
veterinary-surgeon, I learned the diseases among cattle were
not so numerous; the most prevalent were sore throats,
colics, and obstructions of the bowels. The foliage was very
backward.
" May.?This month came in with a very low atmospheric
pressure, and a temperature barely temperate, with the nights
very cold and frosty. The range of the barometer was 1*4 ;
the fall of rain 1*35 inch. The greatest fall of rain took place
on the day and night of the 24th, amounting to '67 of an
inch. With the exception of the first week, the temperature
of the month exceeded that of last May. On the 31st, the
temperature rose to 81?; it has not been so high the last seven
years. The range during the month was 46?. Although the
month was very variable, we had but few diseases under
notice. No epidemic occurred. The most prevalent complaints
were catarrhal affections, influenza, and bronchitis. Several
cases of ague occurred in the labouring classes, arising more from
working in wet clothes and imprudence than from any other
cause. Only two cases of remittent fever came under notice
during the month. Diarrhoea was more prevalent; and one
case terminated in typhoid fever. The casual diseases which
came under treatment in the month were?hepatitis, delirium
tremens, ascites, neuroses, colonitis, phthisis pulmonalis, phleg-
monous inflammation of the leg, dyspepsia, tic douloureux,
amenorrhoea, and furunculus. The cattle were very healthy for
the month.
Record of Deaths in Twenty-one Parishes (population 9,574) from
the Registrar"1 s Book.
March.
Typhus
Bronchitis
Hepatitis
Tetanus
Rupture of bladder
Dropsy
Debility
Decay of nature ...
10
April.
Erysipelas  1
Consumption  1
Bronchitis   3
Croup   1
Quinsy  2
Cerebritis   1
Dropsy   1
Debility    5
Decay of nature .... 2
17
May.
Diarrhoea ....
Consumption ..
Bronchitis ....
Disease of heart
Convulsions ..
Hydrocephalus
Cerebritis ....
Diseased liver
Dropsy
Debility
Decay of nature.
22
Dudley.?Mr. Houghton writes : " The deaths in Dudley in
the last quarter were 287. The average of the last ten corres-
ponding quarters was 254. Hence, the mortality is 33 above
LOCAL REPORTS. 197
the average ; 56 deaths arose from zymotic diseases, being 1 on
5-125 of the whole. This is less then the average of zymotic
deaths; 7 deaths were caused by fever, 4 by scarlet fever,
1 by measles, 2 by small-pox, 11 by croup, 1 by erysipelas,
13 by diarrhoea, 17 by infantile convulsions. Only four of
the cases of croup were certified; and I feel little doubt that
a number of the non-certified cases of croup were, in fact,
diphtheria. The most remarkable feature, however, in the
health of Dudley lately, has been what I may almost call an
epidemic of uterine haemorrhage. Within the last six weeks,
in four practices in Dudley, no fewer than 22 cases of serious
uterine haemorrhage have occurred, and 4 of these have
proved fatal, 11 alarming, and 7 serious. In one case the
placenta was presenting, actively in 7, partially in 8. The
flooding was post parturn; in 2 it was accidental, and in
3 connected with abortions. The particulars of the other case
are not known to me, except that it was severe. Other cases
have no doubt occurred in the other five practices in Dudley,
of which I know nothing. But the occurrence of 22 cases of
haemorrhage, in Dudley, in less than half the practices of the
town, is a remarkable and alarming fact, and one which I am
totally at a loss to account for."
Barrowden. ? Mr. Swann says: "The district upon the
whole has been more healthy this quarter. I have had several
cases of ague in the neighbouring villages. At Ketton, a
large village, four miles east of Barrowden, small-pox has been
raging with unabated fury. Nine deaths have occurred ; and
as far as I can hear, none of the patients had been vaccinated.
Numbers have had it who had been vaccinated, and several
have the disease now, but I hear of no deaths. Scarlet fever,
too, has been fatal in several cases, at the same place; I have
had a few cases of measles in my district."
Bur ton-on-Trent. ?Dr. Thomson states : " That the quarter,
in his district, has been marked by the absence of the com-
moner epidemic diseases. Dropping cases of scarlet fever and
of small-pox liave occurred in the villages, but not with suffi-
cient frequency to be called epidemic."
Lincoln.?Mr. Lowe writes : "We have had a large number
of cases of ague during the whole of this quarter. Diphtheria
has been prevalent the last two or three weeks. We have been
unusually free from typhus fever."
Gainsborough.?Dr. Mackinder writes : "The epidemics have
left us, and our town no longer suffers by a comparison with
the districts reputed healthy. Indeed, we have had reason to
congratulate ourselves, as far as fatal diseases are concerned,
198 PROGRESS OF EPIDEMICS:
for several years past. The recent visitation of throat affection
has been severe, if the number of people who have suffered
from it be the only index ; for scarcely an individual has
escaped ; but the mortality therefrom has been infinitesimally
small. Out of about four hundred cases for which I have pre-
scribed in my own practice, there has been but one death, in
an infant who was quickly asphyxiated by the membranous
exudation. The toxic condition of the atmosphere generating
diphtheria seems as yet to have existed in a few localities only,
and with varying degrees of attenuation. With us the poison
has been diffused in moderate doses, the disease having been
generally mild, but often very persistent. Since my last
report, I have observed many cases of diphtheria, which were
accompanied, as previously stated, by a rash, identical with
scarlatina, yet unlike that disease in the period of its departure,
the eruption in some cases having reappeared daily for two,
three, or four weeks. These certainly may have been cases of
diphtheria engrafted on and modifving the character of scarla-
tina ; but they have not been like those genuine cases of
scarlatina which seem to participate in the epidemic and
exhibit the characteristic fibrinous exudation in the throat,
without manifesting any change in the cuticular efflorescence.
In many of the cases that I attended, both with and without
the rash, the fauces were simply congested and rubeolated ; in
others there was a stratum of inspissated mucus ; while a third
series showed the deposition of a most tenacious and mem-
branoid material. Ulceration of the mouth was frequently co-
existent, but scarcely any of the cases assumed the aspect of
the common ulcerated throat. In many instances the tendency
to bleed was so great, that the surgeon, except under the most
urgent circumstances, would not have been justified in using
the knife. To the last the epidemic maintained its adynamic
type. Close on the heels of diphtheria came scarlatina, of
which we have had many cases of the mildest form, and chiefly
among the children in the workhouse. In all these convales-
cence was rapid. Diarrhoea and dysentery have been far from
infrequent among children, many of whom also suffered from
bronchitis and hooping-cough. Rheumatism and ague have
been more than usually prevalent; of the latter, all the cases
but one that came under my care were introduced. We have
had two cases of lockjaw; one in a middle aged, respectable
man of robust constitution, after an incised wound through
the nail of his left thumb. The wound went on well; but on
the ninth day after the receipt of the injury, he felt a stiffness
in the jaw, and six days afterwards he died, notwithstanding
LOCAL REPORTS. 199
the lobelian saturation of a notorious and ominous char-
latan.* The other patient was a little boy seven years old,
of hereditary predisposition ; his maternal grandfather and
two of his maternal uncles having died of traumatic tetanus.
The exciting cause in this case was an abraded chilblain, and
though the muscles of the jaw, spine and flexors of the toes
were in a state of tonic spasm for several weeks, he got quite
well. 173 paupers were attended as fresh cases in the quarter
?71 in March, 62 in April, and 40 in May. Of these 16 were
between 60 and 70 years old ; 20 between 70 and 80 years
old; 9 between 80 and 90 years old; and one, who re-
covered from an attack of diarrhoea, had arrived at the good
old age of 92. Mr. Chapman, V. S., informs me that throat
affection has been common among horses in this neighbour-
hood since the beginning of May, and though generally amen-
able to treatment, some few cases have been lost from super-
venient pleurisy. The pathological condition in all, as ob-
served at the necropsy, was, besides the effusion into the
pleural cavity and the pulmo-costal adhesions, either a thick
coating of tenacious mucus or an absolute lining with false
membrane of the tonsils, fauces, trachea, and larger bronchial
ramifications. The amount of deposit in some cases was so
great as to induce croupy respiration, and in one instance life
had to be sustained for many days by enemata of milk. Mr.
Chapman gave a bottle and a half of wine daily to one horse,
and believes the disease to be decidedly adynamic. Where ill-
informed men have been allowed to blood the horses, death has
been the result. Parotitis has been a frequent accompaniment.
The prevailing disease among cattle has been influenza. Pleuro-
pneumonia is now uncommon, and the cases that do occur are
curable. Fine weather prevailed throughout the month of
March. Snow fell to the depth of three or four inches in the
first week, and a few showers from the 12th to the 14th. The
first week in April was showery, and from the 24th to the end;
the remainder of the month remarkably fine. The whole of
May was showery. Rain fell on seventeen days out of twenty-
six. The total amount of rain which fell in March was 1^1
inch ; in April, 1*66 ; in May, 2*44 inches. Very little ozone
was exhibited in March, but it was developed freely from the
* Like its sister county, Kutland, with the " Old woman of Wing", Lincoln-
shire is proverbial for its quackery, its bone-setters, its cobbler Coffinites, its
Skeltonites, its dotage-stricken herbalists, its wind pills, its Godfrey's cordial,
its diascordium, and its other soporific poisonous nostrums, with which lazy,
thoughtless, and unfeeling mammas stupify, stultify, and nullify their ne-
glected and truly wretched children.
gOO PROGRESS OF EPIDEMICS
Treatment
commenced.
Feb.20
27
28
Mar. 1
2
3
4
5
6
7
8
9
10
11
12
13
141
15
16
17
18
3.9
20
21
22
23
24
25
26
Diseases of Private Patients.
Pleurodynia, stomatitis ulc., tonsillitis
Faucitis, urticaria
? parotitis
? dysentery, catarrh
? cong. of brain, dysen., diarr.
Diarr., canc. uteri, hoop, cough, dysp.
? epilepsia
Dysentery
Diph.* with erysip., scarlatina, anaemia
Lockj aw
Faucitis, dysentery, cardialgia, colic
? with tonsillitis, pneum.,dysen.
Diphtherite, bronchitis, tertian ague*
Dysentery
? diphtherite, faucitis, stomatitis
Faucitis
? with tonsillitis and rash, dysen.,
stomatitis ulcerosa
Lumbago, bronchitis, dysen., anremia,
convulsions from dentition
Tonsillitis
Quotidian ague, diarrhoea, bronchitis
Faucitis, pleurodynia, conjunctivitis,
tertian ague\
Bronchitis, hooping cough, anasarca
Dysentery, oxaluria
Faucitis, stomatitis, diarrhoea
? ? scarlatina, scabies
Diphtheria, diarrhoea, rheumatism
? faucitis, tonsillitis, scarl at.,dysen.
? diarrhoea, conjunctivitis, dysen.,
ecthyma, epilepsy
Diseases of Pauper Patients.
Anasarca
Conjunctivitis
Rheumatism
Dyspepsia, bronchitis
Gingivitis
Hooping cough, dysentery, colic
Catarrh, bronchitis, frosthite?
? diarrhoea, rheumatism
5) >?
>?
Bronchitis
Anasarca
Catarrh, rheumatism
Pleurodynia, neuralgia, menorrhagia,
Diarrhoea [bronchitis
Colic
Faucitis, vertigo, diarrhoea, frostbite
Paralysis, bronchitis
Rheumatism
Scarlatina
Hremorrhoids, scarlatina||
Scarlatina,diarr.,leucorr.,colic,anasarc.
? pustular ophthalmia
Barometric
pressure at
the temp, of
32? Fahr.
30*268
30-092
29*873
29-778
29-891
29-887
29 898
29-682
29-028
29-167
29-200
29-457
29 731
29-908
30-151
29-288
29-543
29811
29-926
30-158
30-123
30-237
30-367
30-522
30-571
30-519
30-197
30-250
30-220
Temperat.
Dry
bulb.
36
33
36
30
33
34
30
36
34
35
28
34
37
28
33
45
47
46
48
45
50
49
49
51
44
47
51
43
40
Wet
bulb.
32
31
35
30
32
32
29
34
29
32
27
33
32
26
32
43
42
43
45
42
48
46
46
47
42
45
47
39
37
Direction
of Wind.
E. NE.
NE.
NE.
N.
NE.
NE.
NE.
N.
N.
NW.SW.
NW.
NW.
NW.
N.
NW.
sw.
w.
w.
NW.
W.
W.
W.
s.
s.
N.
s.
w.
N.
N.
Rain in 24
hours.
?0
.01
?0
?0
?0
?0
?07
?07
?0
?15
?0
?0
?0
?0
?10
?03
?U
?0
?0
?02
?0
?0
?0
?0
?o
?0
?0
?o
?o
Ozone.
(Schoen.)
2
4
10
10
Remarks.
Clear
Overcast
2 in. snow
Snow shower
Boisterous
2 in. snow
Clear
?
Variable
?
?
Clear
-i
Overcast
Clear
?
?
??
LOCAL REPORTS. 201
Faucitis, bronchitis, colic
Scarlatina, urticaria, dysentery
Faucitis
? diarr., dysen., epilepsy, bursitis
? ? pleurodynia, rheumatism
>>
? cardialoia [meningitis spinalis
Diphth.,monomania,catarrh.pleuritis,
Faucitis, hoop.cough, bronchitis, dysen
? diphtheritis, scarlatina, m)opia
? bronchitis, diarrhoea
? stomatitis, dysentery, dysuria
? bronchi., urticaria, erysip.,pneum.
? hoop.cough, suppressio mensium
? periostitis, diarr., hydrothorax
Apoplexy, paralysis
Erysipelas, pleuritis, catarrh, pleuro-
dynia, convulsions from dentition
Diphth., erysip., faucitis,diarr., dysen.
Faucitis, bronchitis, hooping cough,
rheumatism, colic
Diphtheritis, scarlatina, bronchitis,
meningitis spinalis
Faucitis,epistaxis,leucorr.,monomania
" hoop.cough, catarrh, diarr., leucor.
Dysuria, menorr., bronchit., paralysis
Diphth., paronychia, con v. from teeth.
Rheumatism, prostatis, hydrocepha-
lus, hooping cough
Faucitis, colic, dyspepsia, hoop.cough
? diphtheria,bronchi.,diarr.,syphilis
Faucitis, dysentery
Dysentery
? gravel, congestion of brain
F, c 7 em a, lich en, rh eum ati sm ,di arrh GBa
Phthisis
Scarlatina, dysen., lepra, icterus, oph-
Dysentery [thalmia
Eczema, diarrhoea
Tertian ague If
Faucitis, scarlatina, bronchitis
Hcemorrhoids
Faucitis
Scarlatina, catarrh, conjunctivitis
Tertian ague,diarr.,impetigo,bronchit.
Faucitis, scarlatina, bronchitis, pust.
? [ophthalmia
? furuncle
Scarlatina, frostbite
Colic, scarlatina, struma
Tertian ague, dysuria
Faucitis, diarrhoea
Valvular disease of heart
Rheumatism
Faucitis, scarlatina
Anasarca, pleurodynia
Dysuria, bronchitis, carcinoma of
stomach
Leurorrhoea, dyspepsia, amenorrhcea
Erythema, scarlatina, anasarca
Gingivitis
[mesent.
Diarrhoea, sclerotitis, scarlatina, tabes
30-175
30114
29 995
29fifi3
29-263
29293
29-454
30-003
30-110
29-911
29-873
29 082
29-875
29-942
29 901
29-943
29-979
30-1U
29-939
29-921
29-992
30-202
30-125
30075
30-208
30349
3(K321
29-901
29-955
30096
43
54
50
52
54
39
47
42
44
42
38
35
40
40
44
37
38
41
54
54
47
59
54
53
58
61
60
59.
55
47
40
49
46
47
5l)
38
46
39
40
38
36
35
35
36
40
32
32
38
48
51
46
54
47
48
50
55
54
52
51
45
NW.
SE.
SW.
R.
SW.
N.
SE.
NW.
E.
E.
SE.
SE.
E.
N.
N.
NW.
NW.
SE.
S.
SW.
N.
SE.
S.
NW.
NW.
W.
SE.
SE. W.
SE.
N. S.
?0
?0
?0
?0
?63
?0
?13
?15
?0
?0?
?82
?10
?0
?0
?0
?0
?o
?0
?01
'0
?30
?0
?0
?0
?0
?0
?0
?0
?05
?0
?0
.3
C
10
10
3
10
4
10
10
5
6
10
Scud
M
Showery
?
variable
?
? [&SDOW
Storm of rain
Snow shower
Fine till 18th
Clear till the
24th, and va-
riable to end
of month
* As in my former report, T have confined the term diphtheria to the case in which a deposit of lymph took place. + Ague imported.
t The ague patient came from the fenny district a few weeks before. ( All the cases of frostbite occurred to children in the workhouse.
II Several cases of scarlatina occurred in the workhouse, all of the mildest foim. IT Ague imported from Heckington Fen.
202 PROGRESS OF EPIDEMICS:
Treatment
commenced.
Apl.27
28
29
30
May 1
2
3
4
5
6
7
8
9
10
11
12
13
14
15
16
17
18
19
20
21
22
23
24
25
26
27
28
Diseases of Private Patients.
Diphtheria
Hemorrhoids, bronchitis, jaundice
Vaginitis
Hemiplegia, diarrhoea
Rheumatism
Dyspepsia, diarrhcea, leucorrhcea
Diarrhoea
Menorrhagia, diarrhoea, laryngitis
Aphtha, neuralgia
Faucitis, catarrh
Bronchitis
Diarrhoea
Faucitis
Dropsy
Melaena
Chlorosis, Faucitis
Pleurodynia
Diarrhoea, pustular ophthalmia
Faucitis, sciatica, rheum at., meningitis
Vaginitis, bronchitis, dysentery
Rheumatism, faucitis
Pneumonia
Epilepsia
Faucitis, hoop, cough, haemorrhoids
Catarrh
Bronchitis,hoop.cough,diarr., hysteria
Icterus
Tonsillitis
Diseases of Pauper Patients.
Lumbago, nephritis, paralysis
Scarlatina*
" hooping cough
Constipation, dysentery
Impetigo, febricula, bronchitis
Bronchitis
Tonsillitis
Catarrh, diarrhoea
Bronchitis
Cellulitis, impetigo
Catarrh
Valvular disease of heart
Impetigo
Scarlatina
Paralysis
Phthisis, epithelioma, colic
Barometric
pressure at
the temp.of
32? Fahr.
30-000
30*011
29-597
29-003
29-008
29-328
29-457
29-793
29-882
30-312
30-401
30-354
30191
30-184
30-094
29-835
29-079
29072
29-415
29-477
29-789
29745
29-045
29-854
29-714
29-044
29-779
29-862
30-395
30-101
30-005
Temperat.
Dry
bulb.
44
56
49
49
40
48
49
46
55
50
50
55
50
53
50
49
47
51
59
63
57
60
57
60
61
62
63
35
48
54
59
57
Wet
bulb.
43
52
47
4G
43
45
44
43
50
45
45
50
48
47
45
4G
45
4U
53
50
54
56
49
52
56
54
56
48
47
47
55
51
Direction
of Wind.
N.
S.
S.
sw.
S. N.
N.
N. W.
N. S.
SW. N.
N. E.
E.
NE.
NE.
NE.
NE.
NW.
NW.
SW.
sw.
SW.
SW.
SW.
SW. w.
NW. S.
s.
SW.
SW.
SW.
NW.
N. S.
SW.
NW.
Rain in 24
hours.
0
0
08
02
55
24
05
0
0
0
0
15
01
0
0
06
14
04
38
08
0
01
0
01
05
00
09
47
05
0
0
0
Ozone.
(Sclioen.)
10
2
0
4
3
6
7
3
10
3
10
5
10
10
10
10
2
7
5
10
6
8
Remarks.
The weather
was variable
throughout
this month.
Rain fell on
seventeen
days out of
twenty-four.
Thunder
storm
* Three cases of scarlatina in one house in the town.
LOCAL REPORTS. 203
3d to the 8th of April, from the 16th to the 18th, and from
the 24th to the end of the month, and also throughout the
month of May. The spring was early. The first swallow was
seen on the 10th of April; the cuckoo first heard on the 24th ;
and the lilac first seen in flower on the 6th of May. The oak
was in leaf in the middle of May, and the ash not till the end
of the month. The cereal crops and the meadows promise an
abundant produce." A table of the diseases and meteorology
of Gainsborough for three months, is given at pages 200-2.
Liverpool.?During the quarter ending April, 3,700 deaths
were registered in the borough, including 216 sudden and
violent deaths and deaths in hospitals not included in the
weekly reports. This exceeds by 362 the corrected average of
the same quarter of the preceding ten years. Diseases of the
zymotic class were fatal in 993 cases during the quarter,
being 250 more than the corrected average. The deaths from
scarlatina, hooping cough, typhus, and diarrhoea, show a
decrease; those from measles and small-pox are increased as
compared with the previous quarter. Scarlatina declined
from 369 to 254, hooping cough from 191 to 130, typhus
from 173 to 151, diarrhoea from 134 to 43, while measles
were from 20 to 156, and small-pox from 54 to 123; croup
caused 40 deaths, erysipelas 20, syphilis 20, diphtheria 7,
chicken pox 5, mumps 1. From diseases of the lungs the
deaths were 1,228, being about the corrected average; of
these, 411 were from consumption. Of the 123 who died
from small-pox, 38 are said to have been vaccinated.
Warrington.?Mr. Spinks writes: "Scarlatina has been
very prevalent during the last three months, and still continues
so. The great majority of the cases were very mild. Small-
pox was prevalent in March and April; it raged more amongst
the adults of both sexes than the children. I have seen
patients from forty to sixty years of age ; no deaths occurred
in my district. Ague, as usual, appeared in April and May, in
those Irish labourers who were in the fens of Lincolnshire last
harvest. It seems that the miasma of ague lies dormant in
those patients all winter, when they are not in employment.
I have observed for some years, that so soon as those men get
actively employed in the spring, they are certain to have an
attack of this disease/'
Wigan.?Mr. Hussey says : " If I were to judge of the value
of sanitary improvements from the amount and character of
disease this quarter, I should be much inclined to think we
were considerably behind our needs in this respect. Smallpox
has visited us a second time within a short period, and as if it
?04 PROGRESS OF EPIDEMICS:
MORTALITY AND METEOROLOGY OF LIVERPOOL.
Deaths.
Week ending
Scarlet fever
Measles
Small-pox -
Hooping cough
Croup
Influenza -
Erysipelas -
Cholera
Ague -
Diarrhoea -
Typhus
Carbuncle -
Intemperance
Diseases of lungs
Syphilis
Diphtheria
Mumps
Quinsy
Total deaths during each week
Corresponding week, 1857 -
March.
6th.
15
13
8
G
2
90
3
248
221
13th.
17
16
9
11
2
1
16
116
1
284
IU'3
20th.
J8
9
11
11
94
273
222
27th.
15
7
id
9
18
1
1
83
245
230
April.
3rd.
16
8
10
9
4
3
10
IOC
3
271
235
10th.
16
9
y
5
4
6
12
93
2
258
204
17 th.
105
24th.
10
13
285
May.
1st.
17
22
4
5
i
i
i
13
CO
3
2
200
194
8 th.
14
8
10
10
? ?
3
7
10
*2
87
3
2
24,2
241
15 th.
10
24
11
J
4
10
77
1
243
230
22nd.
If)
13
G
8
C
14:
87
? ?
2
200
192
29th.
206
METEOROLOGICAL OBSERVATIONS.
Barometer: Mean -
Dew-point temperature
Thermometer: Highest (mean)
Lowest (mean) -
Mean -
Mean of 12 years
Range
Mean range, ]2 years -
Rain fell on days ....
Rain fell during hours
Amount of rain in inches -
General direction of wind ...
29-76
27-8
39-5
321
35*8
43 3
7-4
8-1
2
3-3
0-23
NE.
29-69
32-2
41-5
32-3
3(5-9
43 0
92
8-3
7
17-0
0*79
NW.
30-11
430
50-2
42*1
46-2
44* 1
8-1
8-9
5
3-7
0-12
WJSW.
29-02
426
5:)6
42-0
47-8
455
11-6
10.1
5
20 9
0-77
SSE.
29-88
33*9
46-7
36-6
41-7
47-3
10.2
9-3
3
32-5
1-04
ESE.
30-01
87-9
51-9
39-0
45-5
47-9
r>-8
10-9
3
23*2
0-51
SSE.
30*22
43-7
61-8
4G-4
541
49-2
15-5
11-7
1
2-5
0-21
var.
29-73
?13-7
56-2
449
50-6
48-3
113
11-2
4
8-6
0-42
var.
30-05
42-5
54-8
438
49-3
50-1
110
124
2
2-8
0*07
NNW.
29-77
47-1
61-3
50-5
55*8
55-2
10-8
116
4
4*0
0-41
var.
LOCAL REPORTS. 205
f
had not had sufficient time to prolong its stay when first it
came, it has this time made up for it. There have been a great
number of cases in Hindley, with a population of eight thousand,
but the mortality is scarcely one per cent, in those previously
vaccinated. Vaccination carefully performed, and due regard
paid to selection of lymph, with re-vaccination every five years,
would in my opinion rid us in time of a loathsome disease. I have
very good reason for saying, that the quality of lymph used
and sent out is highly objectionable in some instances. The
country never looked better than at present, and with the ap-
proach of summer, disease is not so prevalent as it was a few
months past/7
Bolton-le-Moors.?Mr. Pendlebury writes: "The past quarter
has not been remarkable for any particular epidemic, or excess
of sickness of any kind, which may be accounted for by the
absence of sudden alternations of temperature, and the general
fineness of the weather. Scarlet fever has not prevailed ge-
nerally, but in special localities has been rather rife. Smallpox
in a similar way, has been rather more prevalent in some parts.
During the earlier part of the quarter, carbuncles and boils
prevailed rather extensively. Vegetation has progressed very
favourably, and there is every prospect of an abundant and
luxuriant harvest."
York.?Mr. Procter writes: " The past quarter has been by
no means unhealthy, and the amount of disease which has
shown itself has been small. During the latter part, there has
been scarlet fever, hooping cough, and remittent fever, to some
extent. Smallpox is greatly on the decline, but numerous
cases of chickenpox have shown themselves."
West Auckland.?Mr. Todd says: "During the last quarter
measles and continued fever have prevailed ; the former pretty
generally, the latter but partially and sporadically. Three
cases of continued fever have proved fatal in one family,?viz.;
three sons, of the respective ages of ten, twelve, and nineteen.
The house is situated in a marshy and filthy village, and its
interior anything but clean. The cases were characterized by
intestinal complication at first, and in two of the cases, this was
succeeded by severe cerebral complication ; the third died from
thoracic complication. The origin of this fever could not be traced
to anything like contagion ; the patients had not been near any
one in fever, nor did the disease prevail in the place where they
lived. The brother-in-law of those individuals who resided about
a mile from their residence, visited them several times during
their illness, and took the fever. At first, in this case, there
were sickness and vomiting, diarrhoea, and acute tenderness
206 PROGRESS OF EPIDEMICS.
over the abdomen; in about a week those symptoms subsided,
and the brain became affected, and he died on the fourteenth
day from the commencement of his illness, with low muttering
delirium, subsultus tendinum, retention of urine, and obsti-
nately constipated bowels. No cutaneous rash of any descrip-
tion was observable in any of those cases. This last case adds
another proof to the many, which I have observed, of con-
tinued fever originating from paludal, miasmatic, or local
causes, becoming contagious. It is of the utmost importance,
that the error of the Cullenian doctrine should be pointed
out and refuted by facts, since it has, in my opinion, been the
very groundwork of that altercation which has so long and
so very bitterly prevailed upon this subject. The writers on
both sides have endeavoured to support the doctrine of Cullen,
and have been equally impressed with the truth of his erro-
neous definition of continued fever, only warring with each
other in support of Cullen's distinction, and hence those who
from personal observation have been compelled to acknowledge
the origin of continued fever from paludal sources have yet felt
themselves compelled, at the same time, to deny that a fever
having such an origin can become contagious, and thus they
have been driven to split continued fever into a great many
artificial and laboured divisions; indeed, their principal aim
appears to me to have been to avoid simplicity ; like Hudibras
of old, they appear to be able to divide a hair twixt south
and south-west side. The cases of measles have not been
characterized by any particular complication, with the excep-
tion of slight bronchial affection. During the spring and
summer months, we generally see the disease in its pure and
uncomplicated form/'
Lerwick.?Dr. Spence writes : " During the first fortnight
of March there were very heavy snow storms, and the snow
lay on the ground till near the end of the month. After a
week of fine weather, snow fell again in the beginning of April,
but did not lie long. .The remainder of April and the whole
of May were very variable, with much fog, frequent rain, and
a greater prevalence of northerly wind than is usual at the
season. Vegetation has been very backward. The average
development of Ozone was, in March, 6*8; in April, 6 2 ; and
in May, 6*3. By an error in the last quarterly statement
(No. xiii), I am made to say that typhus occurred in several
weeks in December, January, and February, whereas it should
have been dysentery. There was very little disease of the
epidemic class during the quarter. The total number of deaths
was 20, and the average age was 39, there being 1 below 5
SANITARY LEGISLATION. 207
and 4 above 70. But of these, 7 were violent deaths. One
was a case of drowning ; 1 was occasioned by a fall between
a boat and a wharf, by which rupture of some of the abdominal
viscera (probably the liver or spleen) was produced, and col-
lapse speedily ensued; while 5 of the deaths were the result
of an awful tragedy, in which a man, apparently labouring
under a sudden fit of impulsive insanity, murdered his wife
and three of their children, and then committed suicide. If
these be subtracted from the total deaths, the number will be
13, which is less than during the same quarter last year; and
the average age at death will be 46, which is rather high. The
causes of the remaining deaths where medical certificates were
granted were?small-pox, I ; acute bronchitis, 1 ; valvular
disease of the heart, 1 ; general dropsy, 2 ; congestion of brain,
1 ; chronic inflammation of brain, 1 ; hydrocephalus, 1 ;
chronic hepatitis and subacute gastritis, 1 ; obstruction of
bowels, 1 ; ovarian tumour, 1. The first case of small-pox
was imported from Liverpool, and terminated fatally five
hours after the man was landed. The disease has since spread
rapidly."

				

